# Migraine Headache and the Risk of Depression

**DOI:** 10.7759/cureus.31081

**Published:** 2022-11-04

**Authors:** Hussain A Al Ghadeer, Sadiq A Al Salman, Zahr M Alshakhs, Jehad H Alghanim, Abdulelah A Alneamah, Hussain S Almazyadi, Hashem H Alalawi, Murtada I AlHassan, Bashayr S Alsuwailem, Amjad A Albonasser, Hussain I Aljohar, Yazeed M Alhammadi, Fatimah M Almoaibed, Yaqot A Al Ali, Abdullah I Alali

**Affiliations:** 1 Paediatrics, Maternity and Children Hospital, AlAhsa, SAU; 2 Neurology, King Fahad General Hospital, AlAhsa, SAU; 3 Neurology, King Faisal University, AlAhsa, SAU; 4 Emergency Medicine, Al Ayoun Hospital, AlAhsa, SAU; 5 Internal Medicine, Prince Saud Bin Jalawi Hospital, AlAhsa, SAU; 6 Rehabilitation Medicine, Al Sheikh Hasan Al-Afaliq Rehabilitation Hospital, AlAhsa, SAU; 7 Neurology, Albaha University, Albaha, SAU; 8 Internal Medicine, Maternity and Children Hospital, AlAhsa, SAU

**Keywords:** alahsa, saudi arabia, depression, psychological impact, headache, migraine

## Abstract

Background: Migraine is a primary headache and a complicated neurological disorder with sensory and autonomic abnormalities. Many variables, including genetic and psychological ones, contribute to migraine onset and development. Anxiety and depression are typical psychiatric comorbidities among migraineurs. This kind of comorbidity increased migraine chronicity, treatment effectiveness, and the likelihood of additional comorbidities. The purpose of this research was to determine the prevalence of depression among Saudi migraine sufferers in AlAhsa.

Methods: Descriptive cross-sectional research of 101 migraine patients at King Fahd Hospital-Hofuf, AlAhsa, Saudi Arabia from May to December 2021. Depression was assessed by Patient Health Questionnaire which is a reliable tool (PHQ-9). The PHQ-9 measures the presence and severity of depression. Consider sociodemographic, clinical, and individual variations that impact migraine development and prognosis.

Results: The inclusion criteria were satisfied by 94 migraine patients in total, with a mean age of 36.9 ± 9 years and they are predominantly females 75.5%. The majority of the participants (76.6%) were on medication to relieve migraine attacks and only 13.9% reported that >75% of attacks were relieved by medication. Almost all of the patients (96.8%) used to drink coffee and tea. The prevalence of depression and migraine was revealed to be 42.6% mild and 8.5% severe among the participants. Four statistically significant correlations (p < 0.05) were young age, being female, low level of education at higher risk to have depression compared to another group of migraineurs.

Conclusion: A neurological disorder that commonly causes disability is migraine. Numerous studies have shown that mood disorders and migraines are often co-occurring, and these individuals are more likely to have a migraine-related disability. This research has shown that it is beneficial to prevent psychiatric comorbidity by using PHQ-9 as a regular screening tool for migraine patients.

## Introduction

Migraines vary from other headache types by the following symptoms: lasting four to 72 h; unilateral location; pulsing nature; moderate to severe severity; increased by physical activity; and progresses with nausea, vomiting, phonophobia, or photophobia. Aura symptoms may precede migraine headaches [1]. Migraine is also a multifactorial disorder, with hormonal, genetic, environmental, nutritional, and psychological factors, as well as sleep quality, each playing a distinct role [2]. Migraines and headaches are often related to emotional issues. Depression comorbidity is a common psychiatric illness among migraine headache sufferers. According to a meta-analysis of 12 studies [3] the prevalence of depression among migraineurs ranges from 8.6% to 47.9%. Depression in migraineurs is considered a risk factor for chronic migraine headache, resistance to migraine therapies, drug misuse, suicidal behavior connected to psychosocial impairment, and the influence on migraineur quality of life [4-6]. The brains of migraineurs with comorbid depression differ from those of people with just migraine or simply depression. Many migraine neuroimaging studies revealed brain alterations, noted abnormal functions of specific brain regions, and speculated that these regions might contribute to migraine without aura depressive symptoms [7-10]. Recurrent migraine headache episodes, on the other hand, increase the chance of depression [11]. The variations in the symptoms profiles of migraineurs with and without considerable depression were minimal; the incidence of aura symptoms and migraine aggravation by physical activity were more severe in migraineurs with major depression than in those without depression [12]. Cortical spreading depression (CSD) has been proposed as the condition that causes migraine aura [13]. Furthermore, persons who suffer from migraine have a 2.2-4.0 times greater risk of getting depression than the general population [14].

## Materials and methods

The outpatient neurology service department at King Fahd Hospital-Hofuf in AlAhsa, Saudi Arabia, was the site of the descriptive, observational, and correlational study. All patients who visited a neurology clinic from May to December 2021 and were diagnosed with a migraine headache and receiving regular follow-up care. A brief description of the study was given to the participants who had autonomy for rejection. The privacy and confidentiality of results were maintained. The criteria for inclusion were adults≥18 years of age and diagnosed with primary migraine by a physician at least 6 months ago and not having other comorbidities or psychiatric illnesses. The patients were given access to an online or paper-based questionnaire. The questionnaire was divided into three sections: the first section asked about biographical information. The second section included both conventional and alternative treatment options that individuals utilized to relieve migraine attacks. The Patient Health Questionnaire serves as the last component for the measurement of the prevalence of depression using scales that have been validated and translated (PHQ-9) into Arabic [15-16]. The PHQ-9 scale is a nine-item test that evaluates the presence and severity of depression during the last two weeks. The PHQ-9 items are graded on a Likert scale of 0 (not at all) to 3 (nearly every day) for each item. According to the PHQ-9, depression scores were divided into five groups: little or no depression (0-4), mild depression (5-9), moderate depression (10-14), moderate-severe depression (15-19), and severe depression (20-27).

Data analysis

The data were collected, reviewed, and then fed to Statistical Package for Social Sciences version 21 (IBM Corp., Armonk, NY). All statistical methods used were two-tailed with an alpha level of 0.05 considering significance if the p-value is less than or equal to 0.05. As for PHQ-9, the overall score was obtained by summing all discrete scores for the items which ranged from 0 to 24 points. Participants’ classification according to depression severity was applied in reference to the scale documented cut-off points. Descriptive analysis was done by prescribing frequency distribution and percentage for study variables including participants' bio-demographic data, using drugs and other methods to relieve migraine attacks, medical history, and PHQ-9 items, with depression prevalence and severity. Cross tabulation for showing the distribution of participants’ depression severity according to their bio-demographic data was carried out with the Pearson chi-square test for significance and exact probability test for small frequency distributions.

## Results

A total of 94 participants fulfilling the inclusion criteria with clinically diagnosed migraine completed the study questionnaire. Participants' ages ranged from 18 to more than 45 years with a mean age of 36.9 ± 12.4 years old. Exactly 70 participants (74.5%) were females. Single participants were 19 (20.2%) while 70 (74.5%) were married. As for education, 62 (66%) participants were university graduates and 23 (24.5%) had below university level of education. Exactly 50 (53.2%) participants were not employed, 30 (31.9%) were non-healthcare workers or students and 14 (14.9%) were healthcare workers/students. A monthly income of less than 5000 SR was reported among 28 (29.8%) participants, and 30 (31.9%) had a monthly income of 5000-10000 SR. Exactly 16 (17%) participants complained of chronic health problems (Table 1).

**Table 1:** Socio-demographic data of study participants with migraine, Al-Ahsa, Saudi Arabia.

Socio-demographic data	Count	Column N %
Age in years		
< 25	16	17.0%
25-35	30	31.9%
36-45	31	33.0%
> 45	17	18.1%
Gender		
Male	24	25.5%
Female	70	74.5%
Marital status		
Single	19	20.2%
Married	70	74.5%
Divorced / widow	5	5.3%
Educational level		
Below university	23	24.5%
University	62	66.0%
Post graduate	9	9.6%
Job title		
Not employed	50	53.2%
Non-healthcare worker / student	30	31.9%
Healthcare worker / student	14	14.9%
Monthly income		
< 5000 SR	28	29.8%
5000-10000 SR	30	31.9%
10000-20000 SR	27	28.7%
> 20000 SR	9	9.6%
Have chronic health problem		
Yes	16	17.0%
No	78	83.0%

Drugs and methods used by study participants to relieve migraine attacks, Al-Ahsa, Saudi Arabia. Exactly 72 (76.6%) participants reported using drugs to relieve migraine attacks. The attack severity was relieved by less than 25% of its severity among 24 (33.3%) of them, while 23 (31.9%) reported relief of 25%-50%, and only 10 (13.9%) were relieved by more than 75%. As for alternative methods, 33 (35.1%) reported using Islamic therapy (Ruqia, Quran), 30 (31.9%) used traditional medicine (herbs), and 24 (25.5%) used Cupping therapy, while 24 (25.5%) did not use any of them. Among users, attack severity was relieved by less than 25% among 34 (48.6%), while 10 (14.3%) relived by more than 75%. Smoking was reported among 10 (10.6%) participants while 91 (96.8%) had coffee and tea but soft drinks were taken among 29 (30.9%) (Table 2).

**Table 2:** Drugs and methods used by study participants to relieve migraine attacks, Al-Ahsa, Saudi Arabia.

Methods	No	%
Had drugs to relief migraine attacks		
Yes	72	76.6%
No	22	23.4%
Degree of improvement in migraine attacks after having treatments		
< 25%	24	33.3%
25-<50%	23	31.9%
50-75%	15	20.8%
> 75%	10	13.9%
Alternative medicine usage		
Traditional medicine (herbs)	30	31.9%
Chinese acupuncture	5	5.3%
Cupping therapy	24	25.5%
Islamic therapy (Ruqia, Quran)	33	35.1%
Others	13	13.8%
None	24	25.5%
Degree of improvement in migraine attacks after these methods		
< 25%	34	48.6%
25-<50%	13	18.6%
50-75%	13	18.6%
> 75%	10	14.3%
Usage any of the following frequently		
Smoking	10	10.6%
Power and soft drinks	29	30.9%
Tea and coffee	91	96.8%

Distribution of patients’ health questionnaire items among study participants with migraine, Al-Ahsa, Saudi Arabia. Exactly 89.4% of the participants reported experiencing trouble concentrating on things, 85.1% felt tired or having little energy, 81.9% had little interest or pleasure in doing things, and 81.9% had trouble falling or staying asleep, or sleeping too much. Only 31.9% had thoughts that they would be better off dead or hurting themselves (Table 3).

**Table 3:** Distribution of PHQ items among study participants with migraine, Al-Ahsa, Saudi Arabia. PHQ, Patient Health Questionnaire

PHQ-9 items	Not at all		Several days		More than half the days		Nearly every day	
	No	%	No	%	No	%	No	%
Little interest or pleasure in doing things	17	18.1%	53	56.4%	23	24.5%	1	1.1%
Feeling down, depressed, or hopeless	22	23.4%	41	43.6%	26	27.7%	5	5.3%
Trouble falling or staying asleep, or sleeping too much	17	18.1%	37	39.4%	24	25.5%	16	17.0%
Feeling tired or having little energy	14	14.9%	36	38.3%	29	30.9%	15	16.0%
Poor appetite or overeating	19	20.2%	35	37.2%	27	28.7%	13	13.8%
Feeling bad about yourself or that you are a failure or have let yourself or your family down	48	51.1%	21	22.3%	19	20.2%	6	6.4%
Trouble concentrating on things	10	10.6%	47	50.0%	22	23.4%	15	16.0%
Moving or speaking so slowly that other people could have noticed. Or the opposite being so fidgety or restless that you have been moving around a lot more than usual	28	29.8%	40	42.6%	17	18.1%	9	9.6%
Thoughts that you would be better off dead, or of hurting yourself	64	68.1%	11	11.7%	15	16.0%	4	4.3%

**Figure 1:**
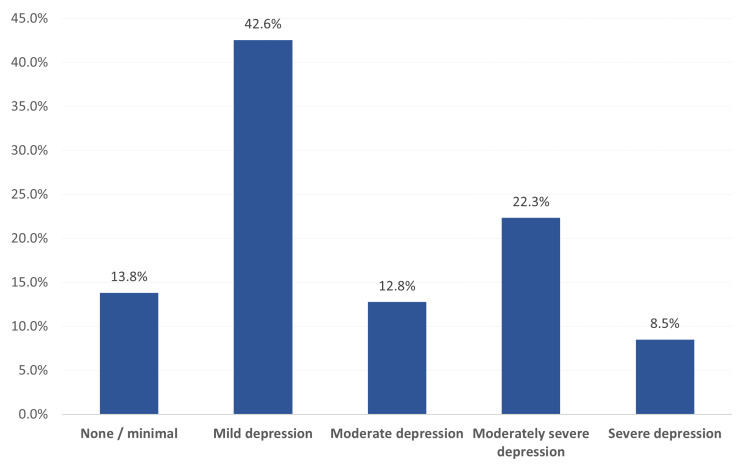
Degree of depression among study participants with migraine, Al-Ahsa, Saudi Arabia.

Degree of depression among study participants with migraine, Al-Ahsa, Saudi Arabia. Exactly 13 (13.8%) of the study participants with migraine had no or minimal depression, 40 (42.6%) had mild depression, 12 (12.8%) had moderate depression, 21 (22.3%) complained of moderately degree depression, and 8 (8.5%) had severe depression (Figure 1).

Distribution of depression severity among participants with migraine by their bio-demographic data. Exactly 68.8% of migraine patients aged less than 25 years had severe depression compared to 23.5% of those who were aged 45 years or more with recorded statistical significance (p=0.030). Also, severe depression was detected among 34.3% of females with migraine in comparison to 20.8% of males (p=0.005). About 39.1% of participants with below university education had severe depression compared to 33.3% of those with post-graduate degree (p=0.002). Severe depression was detected among 35.7% of participants with low income compared to 11.1% of those with the highest income level (p=0.004) (Table 4).

**Table 4:** Distribution of depression severity among participants with migraine by their bio-demographic data. *p < 0.05 (significant)

		Degree of depression						p-value
		None / minimal		Mild / moderate		Severe depression		
		No	%	No	%	No	%	
Age in years	< 25	0	0.0%	5	31.3%	11	68.8%	0.030*
	25-35	5	16.7%	19	63.3%	6	20.0%	
	36-45	5	16.1%	18	58.1%	8	25.8%	
	> 45	3	17.6%	10	58.8%	4	23.5%	
Gender	Male	8	33.3%	11	45.8%	5	20.8%	0.005*
	Female	5	7.1%	41	58.6%	24	34.3%	
Marital status	Single	1	5.3%	8	42.1%	10	52.6%	0.059
	Married	12	17.1%	42	60.0%	16	22.9%	
	Divorced / widow	0	0.0%	2	40.0%	3	60.0%	
Educational level	Below university	2	8.7%	12	52.2%	9	39.1%	0.002*
	University	6	9.7%	39	62.9%	17	27.4%	
	Post graduate	5	55.6%	1	11.1%	3	33.3%	
Job title	Not employed	3	6.0%	27	54.0%	20	40.0%	0.081
	Non-healthcare worker / student	7	23.3%	18	60.0%	5	16.7%	
	Healthcare worker / student	3	21.4%	7	50.0%	4	28.6%	
Monthly income	< 5000 SR	3	10.7%	15	53.6%	10	35.7%	0.004*
	5000-10000 SR	0	0.0%	19	63.3%	11	36.7%	
	10000-20000 SR	5	18.5%	15	55.6%	7	25.9%	
	> 20000 SR	5	55.6%	3	33.3%	1	11.1%	
Diseases	Yes	1	6.3%	12	75.0%	3	18.8%	0.216
	No	12	15.4%	40	51.3%	26	33.3%	
Had drugs to relief migraine attacks	Yes	7	9.7%	41	56.9%	24	33.3%	0.105
	No	6	27.3%	11	50.0%	5	22.7%	
Degree of improvement in migraine attacks after having treatments	< 25%	0	0.0%	16	66.7%	8	33.3%	0.190
	25-<50%	4	17.4%	11	47.8%	8	34.8%	
	50-75%	1	6.7%	7	46.7%	7	46.7%	
	> 75%	2	20.0%	7	70.0%	1	10.0%	
Alternative medicine usage	Traditional medicine (herbs)	6	20.0%	14	46.7%	10	33.3%	0.282
	Chinese acupuncture	0	0.0%	1	20.0%	4	80.0%	
	Cupping therapy	4	16.7%	13	54.2%	7	29.2%	
	Islamic therapy (Ruqia, Quran)	3	9.1%	18	54.5%	12	36.4%	
	Others	3	23.1%	9	69.2%	1	7.7%	
	None	3	12.5%	15	62.5%	6	25.0%	
Usage any of the following frequently	Smoking	3	30.0%	5	50.0%	2	20.0%	0.306
	Power and soft drinks	1	3.4%	19	65.5%	9	31.0%	
	Tea and coffee	13	14.3%	50	54.9%	28	30.8%	

## Discussion

The current study was conducted to explore the psychological impacts of migraine headaches in Al-Ahsa, Saudi Arabia and to detect factors associated with migraine-related psychological factors. People who complain of frequent attacks of migraines are more likely to have mental health issues such as depression and anxiety [17-19]. Persons experiencing migraines are three times more likely to have depression, mainly if the migraine attacks are frequent [20]. Around 30%-50% of people with chronic migraines also develop anxiety, and 20% of those dealing with episodic migraines less than 15 occurrences per month have anxiety, too. There is also some evidence that migraines frequently co-occur with bipolar disorder [21].

The current study showed that depression with different degrees was detected among 86.2% of the migraine-diagnosed participants. It was mild depression among 42% and severe among 30.8% of the participants. The most reported mental health issue among participants was that they experienced trouble concentrating on things, feeling tired or having little energy, having little interest or pleasure in doing things, experiencing trouble falling or staying asleep, or sleeping too much. All were reported among more than three-quarters of the participants with migraine. As for determinants of depression among migraine patients, the study showed that young age, female gender, low educational level, low economic status, and separation were the most significant factors. A lower prevalence of depression was estimated by Dueland et al., who conducted a multinational study and found that 44% of adult females felt depressed because of their migraines [22]. Feeling of frustration due to migraine ranged from 32% in Italy to 84% in Finland; anxiety ranged from 21% in Norway to 57% in Italy, and feelings of confusion ranged from 13% in Greece to 61% in Italy. Also, Yalinay Dikmen et al. estimated that the most common illness among migraine patients was anxiety disorder (38%), followed by depressive disorders (26%), which is much lower than the estimated prevalence by the current study [23]. Jeyagurunathan et al. found that migraine headache was significantly associated with major depressive disorder [prevalence ratio (PR=1.8), bipolar disorder (PR=3.55), generalized anxiety disorder (PR=2.04), and obsessive compulsive disorder (PR=2.20)] [24]. Amouroux and Rousseau-Salvador, reviewed the literature to test for this relationship and concluded that most of the population studies show slightly higher scores on at least one of the anxiety or depression scales in the migraine group as compared to the control group [25]. However, the average score on anxiety and depression among children with migraines was below the pathological level, according to the norms established by the validated scales [14]. Therefore, specific authors use the term "sub-clinical.” Merikangas et al. assessed depression among adults in Zurich and stated a strong association between migraine and depression [odds ratio (OR) 2.2; 95% confidence interval (CI) 1.1-4.8], with an even stronger association between migraine and anxiety disorders (OR 2.7; 95% CI 1.5-5.1) [17]. Hamelsky and Lipton and Peterlin et al. found that persons with migraine had two to five times more likelihood of developing a depressive or anxiety disorder [20, 26]. With adjustment for the other chronic conditions, migraine continued to be significantly related to all psychiatric disorders compared to those without migraine, which is consistent with many other studies [27-28].

As for methods used to relieve migraine attacks associated with pain, the current study showed that three out of each four participants with migraine used drugs with reported low relief percent (<50%). Also, nearly one-third of them used herbs, Islamic therapy for relieving the pain, besides drugs. Cupping therapy was used by one-quarter of the participants, and many of them used more than one method. About half of those participants reported poor attack severity improvement with the methods used.

Limitations

Our study's findings have certain limitations. First, as our data were cross-sectional, we were unable to evaluate the direction of the relationship between depression subtypes and migraine intensity. To evaluate the chronological sequence between the development of depression and migraine, longitudinal investigations are necessary. Another limitation is the relatively small number of sample size. They vary in gender proportions as the majority of the participants were females; thus, future research should include more men. There are also variances in comorbidity, disability, pain severity, and frequency of episodes. We did not make a distinction between episodic and chronic migraine, or between migraine with and without aura. Further retrospective and bidirectional research with a large sample size are recommended to investigate this disparity.

## Conclusions

The current study established that migraine headache is a significant disorder associated with a higher occurrence of psychiatric disorders, especially depression and anxiety. Drug-induced pain relief was poor and alternative methods were also used with no satisfactory relief of pain attacks. These findings can help to create awareness and encourage comprehensive synchronized methods for the management of migraine in healthcare settings. Migraine-associated depression was higher among females, youth, and low socio-economic categories.
